# A New Diterpene Glycoside from *Stevia rebaudiana*

**DOI:** 10.3390/molecules16042937

**Published:** 2011-04-04

**Authors:** Venkata Sai Prakash Chaturvedula, Indra Prakash

**Affiliations:** The Coca-Cola Company, Organic Chemistry Department, Research and Technology, One Coca-Cola Plaza, Atlanta, GA 30313, USA

**Keywords:** *Stevia rebaudiana*, Compositae, Asteraceae, diterpenoid glycosides, spectral data

## Abstract

From the commercial extract of the leaves of *Stevia rebaudiana*, a new diterpene glycoside was isolated besides the known steviol glycosides including stevioside, rebaudiosides A-F, rubusoside and dulcoside A. The new compound was identified as 13-[(2-*O*-β-d-glucopyranosyl-3-*O*-β-d-glucopyranosyl-β-d-glucopyranosyl)oxy] *ent*-kaur-16-en-19-oic acid-(2-*O*-α-l-rhamnopyranosyl-β-d-glucopyranosyl) ester (**1**) on the basis of extensive spectroscopic (NMR and MS) and chemical studies.

## 1. Introduction 

*Stevia rebaudiana* (Bertoni) Bertoni is a perennial shrub of the Asteraceae (Compositae) family native to Brazil and Paraguay, which is often referred to as “the sweet herb of Paraguay”. The major constituents in the leaves of *S. rebaudiana* are the potently sweet diterpenoid glycosides stevioside, rebaudiosides A and D, and dulcoside A. These compounds, which are known as Stevia sweeteners, are glycosides of the diterpene steviol (*ent*-13-hydroxykaur-16-en-19-oic acid) [1]. As a part of our continuing research to discover natural sweeteners [2,3], we have reported several diterpene glycosides from the commercial extract of *S. rebaudiana*. In this article, we present the isolation and structure elucidation based on extensive spectroscopic (NMR and MS) and chemical studies of the new diterpenoid glycoside **1**, identified from the stevia extract (SG-95) obtained from Pure Circle (Kuala Lumpur, Malaysia). 

## 2. Results and Discussion 

**Figure 1:**
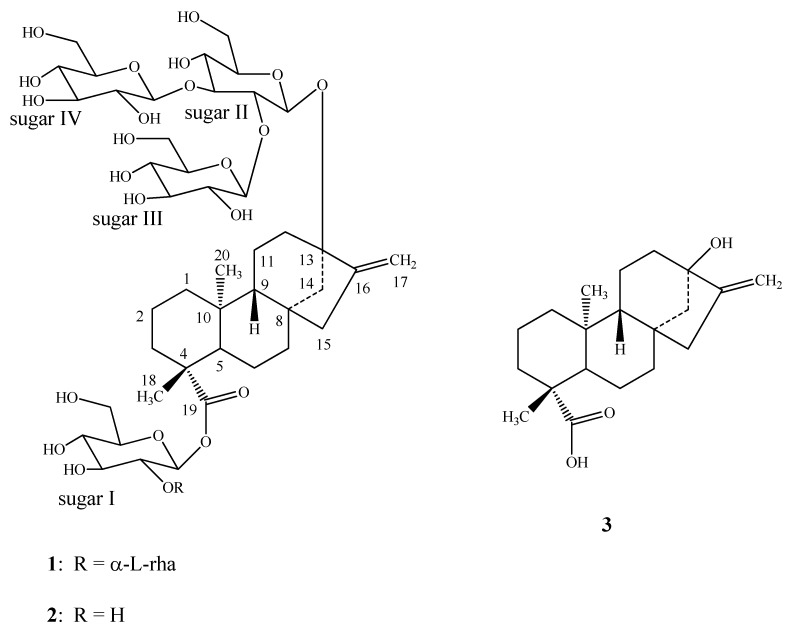
Structure of **1** and other compounds.

Purification of the commercial extract SG-95 obtained from the leaves of *S. rebaudiana* resulted in the isolation of the new diterpenoid glycoside **1**, and the known steviol glycosides, stevioside, rebaudioside A (**2**), rebaudiosides B-F, rubusoside and dulcoside A (Figure 1). The structures of all the known compounds were identified by comparison of their retention times with authentic standards using the HPLC-MS method reported earlier [4] and the spectral data reported in the literature [4,5,6,7,8,9,10,11]. 

Compound **1** was isolated as a colorless oil and its molecular formula was deduced as C_50_H_80_O_27_ on the basis of its positive ESI mass spectrum, which showed an [M+H]^+^ ion at *m/z* 1,113.4977, together with [M+NH_4_]^+^ and [M+Na]^+^ adducts at *m/z* 1,130.5243 and 1,135.4805, respectively. This composition was supported by ^13^C-NMR spectral data. The ^1^H-NMR spectrum of **1** showed the presence of two methyl singlets at δ 0.94 and 1.26, two olefinic protons of an exocyclic double bond as singlets at δ 4.87 and 5.25, nine methylene and two methine protons between δ 0.85–2.27 characteristic for the *ent*-kaurane diterpenoids isolated earlier from the genus *Stevia* [7,8,9]. The basic *ent-*kaurane diterpenoid skeleton was supported by COSY (H-1/H-2; H-2/H-3; H-5/H-6; H-6/H-7; H-9/H-11; H-11/H-12) and HMBC (H-1/C-2, C-10; H-3/C-1, C-2, C-4, C-5, C-18, C-19; H-5/C-4, C-6, C-7, C-9, C-10, C-18, C-19, C-20; H-9/C-8, C-10, C-11, C-12, C-14, C-15; H-14/C-8, C-9, C-13, C-15, C-16 and H-17/C-13, C-15, C-16) correlations. The positive mode ESI MS/MS spectrum of **1** showed fragment ions at *m/z* 951, 789, 627 and 465, suggesting the presence of four hexose moieties. The fragment ion observed at *m/z* 951 was further fragmented to an ion at *m/z* 805, suggesting an additional deoxyhexose unit in its structure. The presence of five sugar units in its structure was supported by the ^1^H-NMR spectrum, which showed the presence of anomeric protons at δ 4.62, 4.66, 4.86, 5.31, and 5.62. 

**Figure 2:**
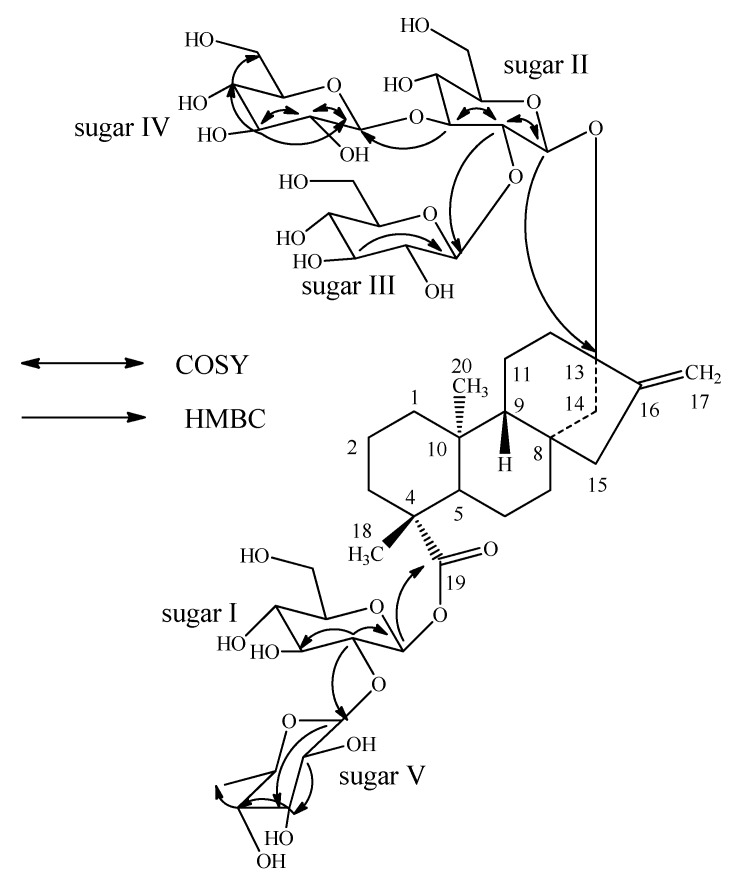
Key COSY and HMBC correlations of **1**.

Enzymatic hydrolysis of **1** furnished an aglycone which was identified as steviol (**3**) by comparison of ^1^H-NMR [10] and co-TLC with standard compound. Acid hydrolysis of **1** with 5% H_2_SO_4_ afforded d-glucose and L-rhamnose, which were identified by direct comparison with authentic samples by TLC [12,13,14]. The ^1^H- and ^13^C-NMR values for all the carbons in **1** were assigned on the basis of COSY, HSQC and HMBC correlations (Table 1). Based on the results from NMR spectral data and hydrolysis experiments of **1**, it was concluded that there are four d-glucose and one l-rhamnose moieties in its structure. A close comparison of the ^1^H- and ^13^C-NMR spectrum of **1** with rebaudioside A (**2**) suggested that compound **1** is also a steviol glycoside which has three glucose residues that are attached at the C-13 hydroxyl as a 2,3-branched β-d-glucotriosyl substituent and another glucose moiety in the form of an ester at C-19 leaving the assignment of the additional rhamnose moiety. The downfield shift for both the ^1^H and ^13^C chemical shifts at C-2′ suggested that the additional rhamnsoe is attached at this position. This was confirmed by the key HMBC correlations: H-2′/C-1′, C-3′, C-1′′′′′ and H-1′′′′′/C-2′, C-2′′′′′, C-3′′′′′ (Figure 2). 

The anomeric proton of the rhamnose residue was observed at δ 5.31 and had a coupling constant of 1.5 Hz, confirming that it has an α-configuration, similar to dulcosides A and B [6]. The large coupling constants observed for the four anomeric protons of the glucose moieties at δ 4.62 (d, *J* = 7.8 Hz), 4.66 (d, *J* = 7.8 Hz), 4.86 (d, *J* = 7.6 Hz), and 5.62 (d, *J* = 7.4 Hz), suggested their β-orientation as reported for steviol glycosides [5,6,7]. Based on the results from chemical and spectral studies, **1** was assigned as 13-[(2-*O*-β-d-glucopyranosyl-3-*O*-β-d-glucopyranosyl-β-d-glucopyranosyl)oxy] *ent*-kaur-16-en-19-oic acid-(2-*O*-α-l-rhamnopyranosyl-β-d-glucopyranosyl) ester.

## 3. Experimental

### 3.1. General

NMR spectra were acquired on Bruker Avance DRX 500 MHz and Varian Unity Plus 600 MHz instruments using standard pulse sequences. The spectra were referenced to the residual solvent signal (δ_H_ 3.30, δ_C_ 49.0 for CD_3_OD), chemical shifts are given in δ (ppm), and coupling constants are reported in Hz. MS and MS/MS data were generated with a Waters Premier Quadrupole Time-of-Flight (Q-TOF) mass spectrometer equipped with an electrospray ionization source operated in the positive-ion mode and ThermoFisher Discovery OrbiTrap in the positive mode electrospray. Samples were diluted with water: acetonitrile (1:1) containing 0.1% formic acid and introduced via infusion using the onboard syringe pump. Preparative HPLC was performed on an Agilent 1100 system using a Phenomenex Prodigy ODS (3) column (250 × 21.2 mm, 5 μm). Semi-preparative HPLC was carried out with a Waters 600E multisolvent delivery system using a Phenomenex Synergi Hydro RP column (250 × 10 mm, 4 μm) column. 

### 3.2. Plant Material

Stevia extract SG-95, the commercial sample consisting of a mixture of diterpenoid glycosides from the leaves of *S. rebaudiana* was obtained from Pure Circle (Kuala Lumpur, Malaysia). A voucher specimen is deposited at The Coca-Cola Company, No. VSPC-3166-002.

### 3.3. Isolation

Preliminary separation of the crude stevioside extract was carried out using a preparative HPLC method employing a water/acetonitrile (B) gradient (25% B for 8.5 min, 25 to 29% B over 1.5 min, 29 to 30% B over 5.5 min, 30 to 34% B over 2.0 min, 34% B for 6 min, 34 to 52% B over 2.0 min, 52% B for 3.0 min, 52 to 70% B over 1.0 min, 70% B for 5.5 min) at 20 ml/min. The baseline fraction at 16.8 min was collected and dried by rotary evaporation under reduced pressure as the crude impurity fraction. Final fractionation was then performed using HPLC method by injecting the crude impurity fraction (over several runs) on a Synergi Hydro RP column by semi-preparative HPLC using a gradient of water (0.01156% acetic acid, 0.02844% ammonium acetate) in acetonitrile (25% B for 8.5 min,25 to 29% B over 1.5 min, 29 to 30% B over 5.5 min, 30 to 34% B over 2.0 min, 34% B for 6 min, 34 to 52% B over 2.0 min, 52% B for 3.0 min, 52 to 70% B over 1.0 min, 70% B for 5.5 min) at 5 mL/min to yield **1** (*t*_R_ 10.6 min, 1.6 mg). All the known compounds were identified in comparison of their retention times with authentic standards using the HPLC-MS method as described previously [4] and the spectral data that were reported in the literature [4,5,6,7,8,9,10,11].

*13-[(2-O-β-d-glucopyranosyl-3-O-β-d-glucopyranosyl-β-d-glucopyranosyl)oxy] ent-kaur-16-en-19-oic acid-(2-O-α-l-rhamnopyranosyl-β-d-glucopyranosyl) ester* (**1**). Colorless film; ^1^H-NMR (CD_3_OD, δ ppm) and ^13^C-NMR (CD_3_OD, δ ppm) spectroscopic data, see Table 1; +ESI TOFMS m/z 1,113.4977 (calcd. for C_50_H_81_O_27_: 1,113.4965).

*Enzymatic hydrolysis of* **1**. A solution of **1** (250 μg) was dissolved in 0.1 M sodium acetate buffer, pH 4.5 (2.5 mL) and crude pectinase from *Aspergillus niger* (50 µL, Sigma-Aldrich, P2736) was added. The mixture was stirred at 50^o^ C for 48 hr. The product precipitated out during the reaction and was filtered and then crystallized from methanol (MeOH). The resulting steviol (**3**) was identical to an authentic sample by TLC and ^1^H-NMR.

*Acid Hydrolysis of* **1**. To a solution of **1** (250 μg) in MeOH (1 mL) was added 5% H_2_SO_4_ (1 mL) and the mixture was refluxed for 8 hours. The reaction mixture was then neutralized with saturated sodium carbonate and extracted with ethyl acetate (EtOAc, 2 × 5 mL) to give an aqueous fraction containing sugars and an EtOAc fraction containing the aglycone part. The aqueous phase was concentrated and compared with standard sugars using the TLC system EtOAc/*n*-butanol/water (2:7:1) and CH_2_Cl_2_/MeOH/water (10:6:1) [12,13,14]; the two sugars were identified as l-rhamnose and d-glucose. 

## 4. Conclusions 

A new diterpenoid glycoside **1**, as well as nine known steviol glycosides – stevioside, rebaudiosides A-F, rubusoside and dulcoside A – were isolated from the commercial extract obtained from the leaves of *S. rebaudiana* obtained from Pure Circle (Kuala Lumpur, Malaysia). The new compound was identified as 13-[(2-*O*-β-d-glucopyranosyl-3-*O*-β-d-glucopyranosyl-β-d-glucopyranosyl)oxy] *ent*-kaur-16-en-19-oic acid-(2-*O*-α-l-rhamnopyranosyl-β-d-glucopyranosyl) ester on the basis of 2D NMR and +EI TOF MS, as well as chemical studies.

## Figures and Tables

**Table 1:** ^1^H- and ^13^C-NMR spectral data (chemical shifts and coupling constants) for **1** in CD_3_OD. ^a^

Position	^1^H NMR	^13^C NMR
1	0.85 (m, 1H), 1.88 (m, 1H)	41.5
2	1.41 (m, 1H), 1.94 (m, 1H)	20.0
3	1.06 (m, 1H), 2.27 (m, 1H)	38.5
4		45.1
5	1.10 (m, 1H)	58.7
6	1.88 (m, 1H), 1.94 (m, 1H)	22.6
7	1.43 (m, 1H), 1.55 (m, 1H)	42.5
8		43.0
9	1.00 (m, 1H)	54.8
10		40.5
11	1.65 (m, 1H), 1.80 (m, 1H)	21.0
12	1.53 (m, 1H), 1.95 (m, 1H)	38.4
13		88.5
14	1.53 (m, 1H), 2.25 (d, *J* = 12.2, 1H)	45.2
15	2.05 (m, 1H), 2.14 (d, *J* = 17.4, 1H)	48.4
16		153.4
17	4.87 (s, 1H), 5.25 (s, 1H)	105.4
18	1.26 (s, 3H)	29.4
19		176.8
20	0.94 (s, 3H)	16.3
1′	5.62 (d, *J* = 7.4, 1H )	93.8
2′	3.59 (m, 1H)	82.6
3′	3.44 (m, 1H)	77.9
4′	3.34 (m, 1H)	71.4
5′	3.36 (m, 1H)	78.2
6′	3.60 (m, 1H), 3.82 (m, 1H)	62.3
1′′	4.62 (d, *J* = 7.8, 1H)	97.2
2′′	3.63 (m, 1H)	79.6
3′′	3.72 (m, 1H)	87.5
4′′	3.38 (m, 1H)	70.0
5′′	3.30 (m, 1H)	77.1
6′′	3.60 (m, 1H), 3.82 (m, 1H)	62.7
1′′′	4.86 (d, *J* = 7.6, 1H)	103.5
2′′′	3.18 (t, *J* = 8.2, 1H)	76.1
3′′′	3.32 (m, 1H)	77.8
4′′′	3.13 (m, 1H)	72.2
5′′′	3.44 (m, 1H)	78.1
6′′′	3.56 (m, 1H), 3.81 (m, 1H)	63.1
1′′′′	4.66 (d, *J* = 7.8, 1H)	104.0
2′′′′	3.26 (m, 1H)	75.1
3′′′′	3.42 (m, 1H)	78.6
4′′′′	3.32 (m, 1H)	71.1
5′′′′	3.36 (m, 1H)	77.8
6′′′′	3.62 (m, 1H), 3.80 (m, 1H)	62.4
1′′′′′	5.31 (d, *J*=1.5, 1H)	101.4
2′′′′′	3.89 (m, 1H)	71.8
3′′′′′	3.63 (m, 1H)	72.0
4′′′′′	3.36 (m, 1H)	73.6
5′′′′′	3.75 (m, 1H)	70.0
6′′′′′	1.24 (*d*, *J* = 6.3, 3H)	18.0

^a^ assignments made on the basis of COSY, HSQC and HMBC correlations; ^b^ Coupling constants are in Hz; ^c^ Chemical shift values are in δ (ppm).
